# Speed Calibration and Traceability for Train-Borne 24 GHz Continuous-Wave Doppler Radar Sensor †

**DOI:** 10.3390/s20041230

**Published:** 2020-02-24

**Authors:** Lei Du, Qiao Sun, Jie Bai, Xiaolei Wang, Tianqi Xu

**Affiliations:** Division of Mechanics and Acoustics, National Institute of Metrology, Beijing 100029, China; sunq@nim.ac.cn (Q.S.); baijie@nim.ac.cn (J.B.); wangxl@nim.ac.cn (X.W.); xutianqi18@mails.ucas.ac.cn (T.X.)

**Keywords:** train speed measurement, speed calibration, Doppler radar sensor (DRS), single-channel, dual-channel, freight train, high-speed train, urban rail train, traceability, Doppler shift simulation

## Abstract

The 24 GHz continuous-wave (CW) Doppler radar sensor (DRS) is widely used for measuring the instantaneous speed of moving objects by using a non-contact approach, and has begun to be used in train-borne movable speed measurements in recent years in China because of its advanced performance. The architecture and working principle of train-borne DRSs with different structures including single-channel DRSs used for freight train speed measurements in railway freight dedicated lines and dual-channel DRSs used for speed measurements of high-speed and urban rail trains in railway passenger dedicated lines, are first introduced. Then, the disadvantages of two traditional speed calibration methods for train-borne DRS are described, and a new speed calibration method based on the Doppler shift signal simulation by imposing a signal modulation on the incident CW microwave signal is proposed. A 24 GHz CW radar target simulation system for a train-borne DRS was specifically realized to verify the proposed speed calibration method for a train-borne DRS, and traceability and performance evaluation on simulated speed were taken into account. The simulated speed range of the simulation system was up to (5~500) km/h when the simulated incident angle range was within the range of (45 ± 8)°, and the maximum permissible error (MPE) of the simulated speed was ±0.05 km/h. Finally, the calibration and uncertainty evaluation results of two typical train-borne dual-channel DRS samples validated the effectiveness and feasibility of the proposed speed calibration approach for a train-borne DRS with full range in the laboratory as well as in the field.

## 1. Introduction

High-speed railway has had a rapid development in recent years in China and the total mileage of high-speed railway passenger dedicated lines has exceeded 35,000 km, accounting for about 70% of the world’s high-speed railways in commercial service. The number of high-speed trains suitable for driving in high-speed railway passenger dedicated lines has exceeded 3000 in China, accounting for about 75% of the world’s high-speed trains, and the highest speed reached by high-speed trains used in commercial service has been up to 400 km/h until now. Meanwhile, many big cities of China have begun to extensively construct railway passenger dedicated lines in urban, suburban, and intercity areas in recent decades. Speed surveillance is a key component of a railway management system for automatic train control in railway passenger dedicated lines to ensure the operation safety and high efficiency of rail transits [1,2,3,4,5]. A train speed surveillance system generally relies on a combination of a track-side fixed subsystem and a train-borne movable subsystem to calculate the instantaneous speed and covered distance of trains in China. Traditional train-borne movable speed measurement approaches of conventional trains usually rely on a single kind of wheel speed sensor including an axle-mounted wheel speed sensor, eddy current speed sensor, or Hall speed sensor, etc. These kinds of wheel speed sensors measure the rotational speed of the train wheel to indirectly calculate the train speed and covered distance. One disadvantage of these wheel speed sensors is that it may lead to large deviations and even no results in train speed measurement when wheel slippage occurs, which may cause a serious impact on train operation safety. Another disadvantage is that these wheel speed sensors measure the train speed by using a contact approach, which may suffer from the problem of excessive wear and tear, especially at high speed.

The China Train Control System class 3 (CTCS-3) is the new generation standard for train control systems in China, and has been applied in many high-speed railway passenger dedicated lines. According to the safety requirements of CTCS-3, the speed controls of the train-borne speed measurement subsystem are specified as sounding an alarm at an overspeed of 2 km/h, triggering service brake at an overspeed of 5 km/h, and triggering emergency brake at an overspeed of 15 km/h in a high-speed railway line that is greater than or equal to 300 km/h. In order to meet the extremely high safety requirements of CTCS-3 in railway passenger dedicated lines, the architecture of the train-borne speed measurement subsystem has changed from a single wheel speed sensor to the fusion of multiple speed sensors, which can take full advantage of redundant data and complementary information of multiple kinds of speed sensors with different working principles. With the advantage of high-accuracy, wide-range, long-lifetime, stability, and reliability [6,7,8,9], the 24 GHz continuous wave (CW) Doppler radar sensor (DRS) has been widely used in high-speed and urban rail trains, working together with some of the other conventional wheel speed sensors to directly measure the instantaneous speed of trains in real time by using a non-contact approach, which is not affected by wheel slip and spin. Unlike the dual-sided symmetrical structure of vehicle-borne dual-channel DRS with the Janus configuration, which is used as a mobile standard speed-measuring instrument for the field verification of traffic speed meters in road traffic [10,11,12], train-borne dual-channel DRSs usually adopt a single-sided asymmetric structure, which is a more common and complicated configuration than the Janus configuration used in vehicle-borne dual-channel DRSs and seeks no approximation methods for the solution on train speed calculation [10]. Additionally, the train-borne single-channel DRS is still widely used for speed measurements of freight trains in railway freight dedicated lines because of its low-cost, long-lifetime, and stability [1].

To ensure the accuracy, reliability, and traceability of train-borne speed measurement results, each DRS must be calibrated before being mounted on the train. Traditional speed calibration methods for train-borne DRSs include the acoustic resonant and the mechanical movement methods. The acoustic resonant method is based on Doppler shift simulation by using tuning forks with different specific frequencies [13,14]. Two main disadvantages of this method are the discontinuous problem of speed calibration points and can only be applied for train-borne single-channel DRSs. The mechanical movement method is based on the relative motion principle by using a moving pavement simulator [15,16]. Two main disadvantages of the mechanical movement method are the limited speed simulation range and the traceability problem.

A new calibration method for train-borne dual-channel DRSs of high-speed train has been proposed based on dual Doppler shift simulation [17]. On the basis of the proposed calibration method in [17], a detailed speed calibration process and realization of a programmable and composable 24 GHz CW radar target simulation system, which is applicable to the calibration of single-channel DRSs as well as dual-channel DRSs, are presented in this paper. The rest of this paper is organized as follows. Section 2 introduces the working principle of train-borne DRSs including single-channel DRSs used for freight train speed measurements in railway freight dedicated lines and dual-channel DRSs used for speed measurements of high-speed and urban rail trains in railway passenger dedicated lines. Section 3 describes the disadvantages of two traditional speed calibration methods and the advantages of the proposed speed calibration method for train-borne single-channel and dual-channel DRSs, and presents the detailed process of the proposed speed calibration method based on Doppler shift signal simulation. Section 4 presents the realization and traceability of a programmable and composable 24 GHz CW radar target simulation system to verify the proposed speed calibration method. Section 5 describes the speed calibration results and uncertainty evaluation results of two typical train-borne dual-channel DRS samples to validate the effectiveness and feasibility of the proposed speed calibration method. Finally, the paper is concluded and future work is given in Section 6.

## 2. Related Works

### 2.1. Architecture of Train-Borne DRS

**Figure 1:**
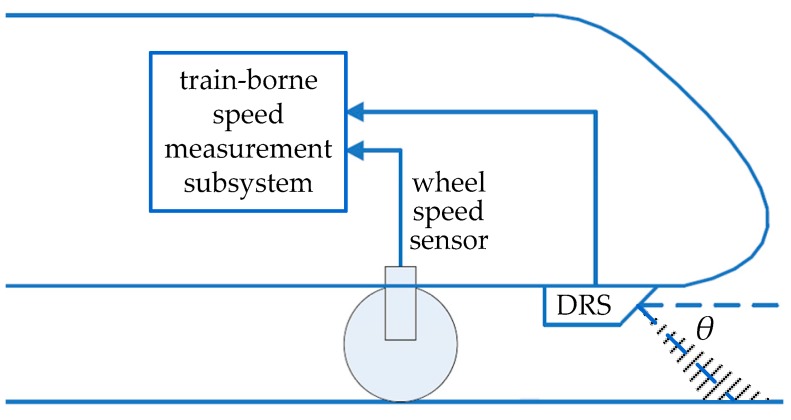
The installation schematic of train-borne DRS.

As shown in Figure 1, the architecture of the train-borne speed measurement subsystem in high-speed and urban rail trains adopts the fusion of DRSs and other wheel speed sensors. It can take full advantage of the redundant data and complementary information of these two kinds of speed sensors with different working principles and improve the accuracy and reliability of train-borne speed measurement results in the railway passenger dedicated lines. At least two train-borne DRSs are respectively mounted at the bottom of the train heads at the front and rear of high-speed trains or urban rail trains, and directly measure the instantaneous train speed in real time by emitting CW microwave signals at a specified angle toward the rail surface based on the principle of the Doppler effect.

### 2.2. Train-Borne Single-Channel DRS

**Figure 2:**
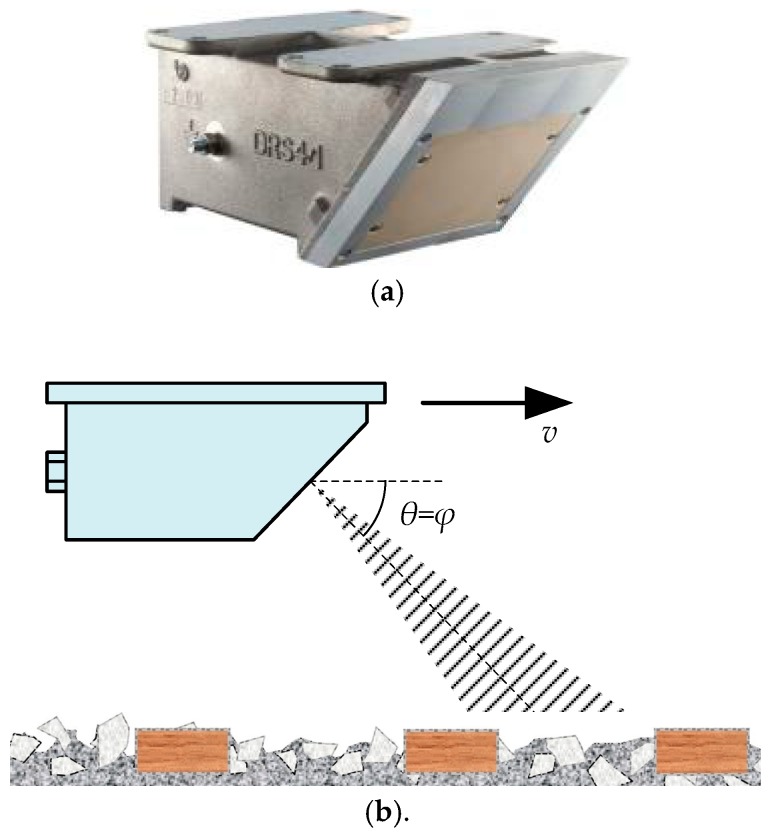
Train-borne single-channel DRS: (**a**) Appearance view of DRS4; (**b**) Schematic diagram of working principle.

Theoretically speaking, a single-channel is enough for train-borne DRS to measure the instantaneous train speed. A typical type of train-borne single-channel DRS product is the DRS4 manufactured by DEUTA-WERKE GmbH, as shown in Figure 2a, which is still widely used for the speed measurement of freight trains in railway freight dedicated lines.

The schematic diagram of a train-borne single-channel DRS is shown in Figure 2b, where *v* is the train speed, *φ* is the design incident angle of train-borne single-channel DRS, and *θ* is the included acute angle between the direction of train travel and a line from the antenna beam direction of DRS to the rail surface (abbreviated to “included angle” hereafter). The working principle of the train-borne single-channel DRS is similar to that of the vehicle-borne single-channel DRS [10] and traffic radar-based speed meters [18,19], which is also based on the principle of the Doppler effect as expressed by Equation (1):

(1) $$v=\frac{C\cdot f_{d}}{2f_{0}\cdot \cos\theta },$$

where *f*_d_ is the Doppler shift; *C* is the electromagnetic wave propagating speed in air; and *f*_0_ is the emitted frequency of the train-borne single-channel DRS. Theoretically speaking, the included angle *θ* is equal to the design incident angle *φ*, and therefore the travel speed value *v* of the train can be calculated according to Equation (1).

**Figure 3:**
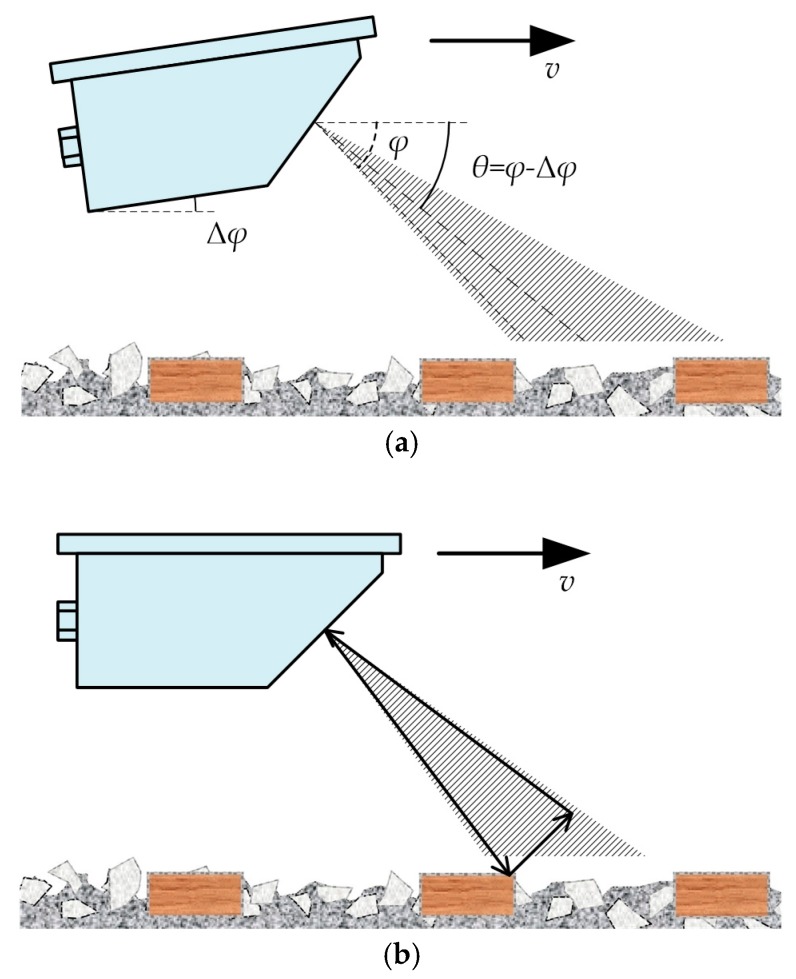
Problems of train-borne single-channel DRS: (**a**) Angle deflection problem caused by the installation deviation and the jolt of motion; (**b**) Multipath effect problem.

However, the train-borne single-channel DRS has the same problems as the vehicle-borne single-channel DRS, as shown in Figure 3. However, angle deflection is obviously inevitable in the actual situation when DRS is mounted on the train, and may also be caused by jolts or vibrations during the train in motion. In this situation, as shown in Figure 3a, the included angle *θ* is no longer equal to the design incident angle *φ*, but can be expressed as *θ* = *φ* – Δ*φ*, where Δ*φ* is the deflection angle. However, Δ*φ* is unknown and even dynamically variable, determined by various factors including installation deviation, jolt, track subgrade, etc. It is also impossible for single-channel DRSs to measure the deflection angle Δ*φ* in real time. Therefore, the single-channel DRS has the inevitable problem of large speed measurement errors in the actual situation. According to the numerical results of a vehicle-borne single-channel DRS, the relative speed measurement error of a single-channel DRS will be more than ±1.0% when the deflection angle Δ*φ* is larger than 0.5° [10], which is an extremely high requirement for installation accuracy and jolt control. Therefore, a single-channel DRS cannot measure the travel speed value *v* of the train with high accuracy because of the unknown included angle *θ* in Equation (1) in the actual situation. For this reason, the single-channel DRS is no longer used for high-accuracy speed measurements of high-speed and urban rail trains in railway passenger dedicated lines, but only for freight train speed measurements in railway freight dedicated lines.

Aside from the problem of the unknown included angle *θ*, there is still the problem of abnormal speed measurement results caused by the multipath effect in a train-borne single-channel DRS [20,21]. In an ideal situation, the received echo signal should reach the receiving antenna of the DRS with the same propagation path as the transmitted signal. In the actual situation as shown in Figure 3b, however, the received echo signal may sometimes use two or more different propagation paths from the transmitted signal to return to the receiving antenna, which is called the multipath effect in radar. The multipath effect may produce multipath echoes caused by the reflection from the concrete sleepers on the subgrade or the stones on the ballasted track. Since the phase difference between the multipath echoes is random, vector superposition of the received echoes is sometimes strong and sometimes weak, which results in a sudden increase or decrease in speed-measuring values and the rapid decline in the speed-measuring performance.

### 2.3. Dual-Channel DRS

The problem of large speed measurement errors in the train-borne single-channel DRS is mainly caused by the unknown and even dynamically variable included angle *θ* in Equation (1), where *θ* is equal to *φ* – Δ*φ* or *φ* + Δ*φ*. Thus, the travel speed value *v* cannot be accurately calculated according to Equation (1) because of the unknown included angle *θ* in the actual situation, and can only be estimated based on an approximation approach of ignoring the deflection angle Δ*φ* [10], which means *θ* is assumed to be identically equal to *φ* in Equation (1). A solution to the large speed measurement error in the single-channel DRS is to add a channel to form a dual-channel configuration of DRS.

#### 2.3.1. Vehicle-Borne Dual-Channel DRS with the Janus Configuration

**Figure 4:**
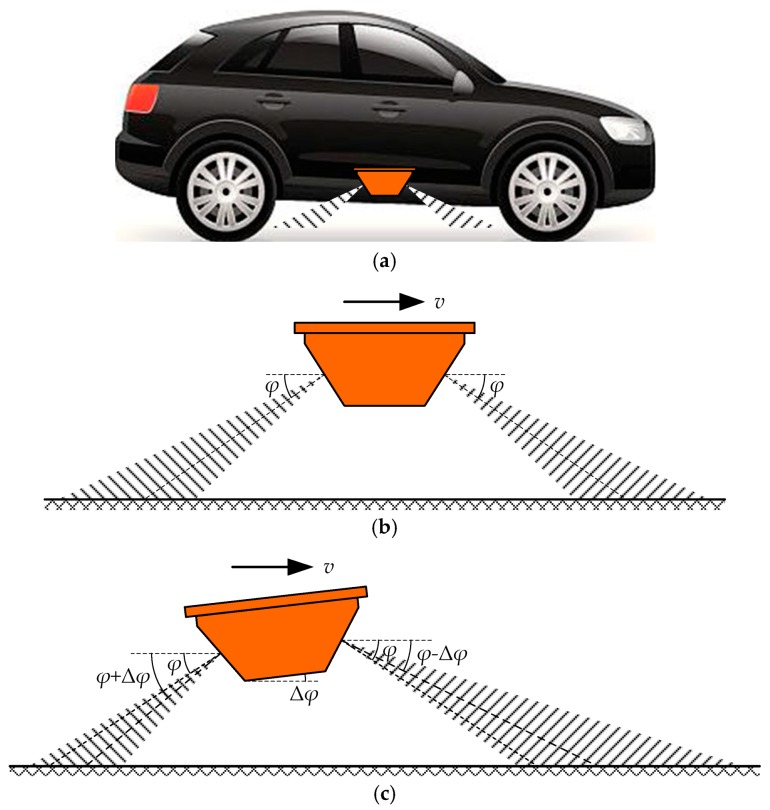
Vehicle-borne dual-channel DRS with the Janus configuration: (**a**) Installation schematic; (**b**) Schematic diagram of working principle in the ideal situation without a deflection angle; (**c**) Schematic diagram of working principle in the actual situation with a deflection angle.

A dual-sided symmetrical structure of a vehicle-borne dual-channel DRS with the Janus configuration, which is used as a mobile standard speed-measuring instrument for the field verification of traffic speed meters in road traffic, has been proposed in [10], as shown in Figure 4.

The dual-channel DRS with the Janus configuration is the most simplified structure of a dual-channel DRS, and has a symmetrical structure and the same signal source. As shown in Figure 4a, a vehicle-borne dual-channel DRS with the Janus configuration is typically mounted at the side of reference vehicle and measures the reference vehicle speed in real time. In an ideal situation without the deflection angle caused by installation deviation or motion jolt as shown in Figure 4b, the vehicle-borne dual-channel DRS with the Janus configuration travels along the road with the speed *v*, and its two antennas of dual-channel respectively point to the road surface in the opposite direction at the design incident angle *φ* with the same emitted frequency *f*_0_. When the deflection angle Δ*φ* exists in the actual situation as shown in Figure 4c, the travel speed value *v* can be respectively calculated by the two channels of the dual-channel DRS with the Janus configuration and can be written by Equation (2):

(2) $$\{\begin{array}{c} v=\frac{C\cdot f_{\mathrm{dr}}}{2f_{0}\cdot \cos\left(\phi -\Delta \phi \right)}\\ v=\frac{C\cdot f_{\mathrm{dl}}}{2f_{0}\cdot \cos\left(\phi +\Delta \phi \right)} \end{array},$$

where *f*_dr_ and *f*_dl_ are the Doppler shifts from the two channels of the dual-channel DRS with the Janus configuration. Thus, the travel speed value *v* and the deflection angle Δ*φ* can be calculated according to the solutions of Equation (2). Therefore, the dual-channel DRS with the Janus configuration can automatically correct the effect of installation deviation or motion jolt on its speed-measuring accuracy and measure its travel speed value *v* with high accuracy in real time. The speed measurement error of the dual-channel DRS with the Janus configuration is smaller than that of a single-channel DRS at the same deflection angle, and also increases much more slowly than that of the single-channel DRS with the increase in deflection angle [10].

However, the vehicle-borne dual-channel DRS with the Janus configuration is not applicable for use in train speed measurements with high accuracy. First, the two channels of dual-channel DRS with the Janus configuration share the same signal source with the same emitted frequency *f*_0_ to simplify the configuration and improve the efficiency. Therefore, the signal interference problem of two channels is inevitable in the dual-channel DRS with the Janus configuration, and may result in a sudden increase or decrease in speed-measuring values once in a while, which cannot meet the extremely high safety requirements in railway passenger dedicated lines. Second, the dual-channel DRS with the Janus configuration can be seen as a combination of two single-channel DRSs pointing in the opposite direction, and therefore cannot solve the problem of abnormal speed measurement results caused by the multipath effect [20,21]. Third, the improvement in the speed-measuring performance of the dual-channel DRS is achieved at the expense of signal processing time. In order to reduce the signal processing complexity and ensure the real-time speed measurement, an approximate method is used to solve Equation (2) on the precondition of a deflection angle Δ*φ* less than 8° in the dual-channel DRS with the Janus configuration [10]. Finally, since the dual-channel DRS with the Janus configuration is a dual-sided symmetrical structure, it can only measure the travel speed value, but cannot determine the direction of travel. The travel direction determination is essential to the speed measurements of high-speed and urban rail trains because the train head can move forwards or backwards in railway passenger dedicated lines.

#### 2.3.2. Train-Borne Single-Sided Dual-Channel DRS

**Figure 5:**
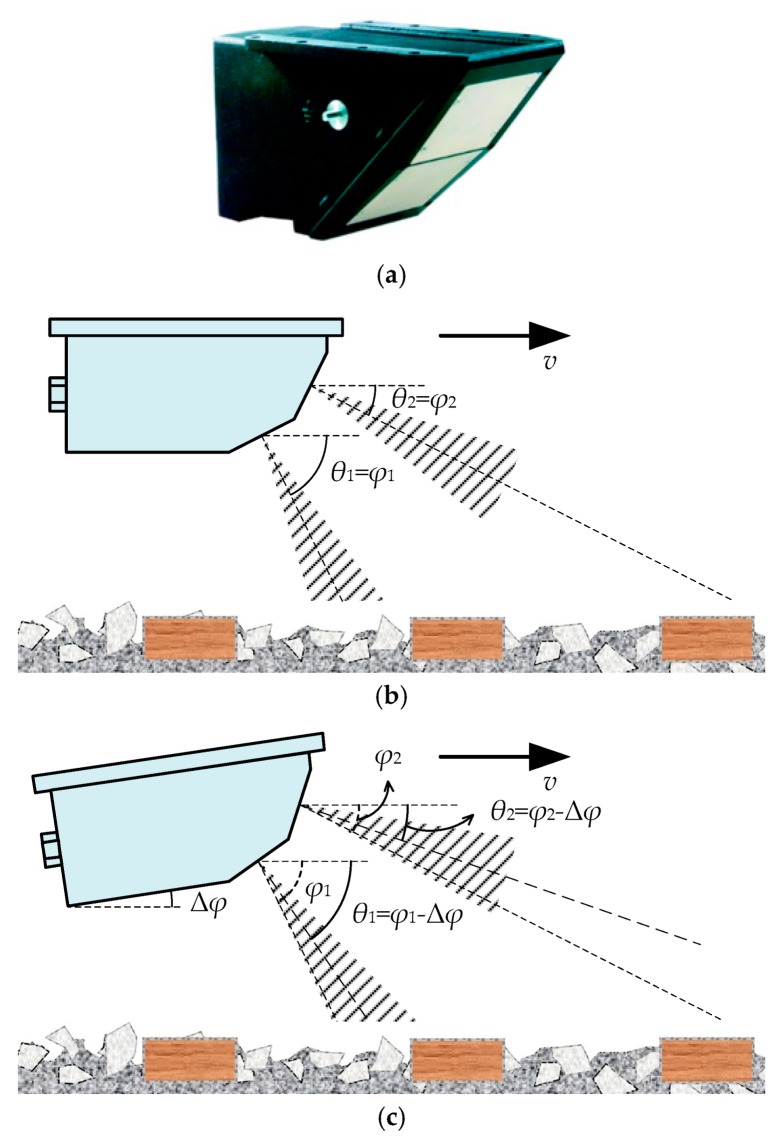
Train-borne dual-channel DRS: (**a**) Appearance view of DRS05; (**b**) Schematic diagram of working principle in the ideal situation without a deflection angle; (**c**) Schematic diagram of working principle in the actual situation with a deflection angle.

In order to make dual-channel DRSs for use in the measurement of train speeds with high accuracy, the train-borne dual-channel DRS usually adopts a single-sided asymmetric structure as shown in Figure 5, which is a more common and complicated configuration than the Janus configuration used in vehicle-borne dual-channel DRS.

A typical type of train-borne dual-channel DRS product is DRS05, which is manufactured by DEUTA-WERKE GmbH, as shown in Figure 5a. In the ideal situation without deflection angle, as shown in Figure 5b, a train-borne dual-channel DRS travels along the railway with the speed *v*, and its two antennas of dual-channel respectively point to the rail surface in the identical direction at the design incident angles of *φ*_1_ and *φ*_2_ with different emitted frequencies of *f*_1_ and *f*_2_. When the deflection angle Δ*φ* exists in the actual situation as shown in Figure 5c, the travel speed value *v* can be respectively calculated by two channels of the train-borne dual-channel DRS, according to the Doppler shift formula and can be written by Equation (3):

(3) $$\{\begin{array}{c} v=\frac{C\cdot f_{d1}}{2f_{1}\cdot \cos\left(\phi_{1}-\Delta \phi \right)}\\ v=\frac{C\cdot f_{d2}}{2f_{2}\cdot \cos\left(\phi_{2}-\Delta \phi \right)} \end{array},$$

where *f*_d1_ and *f*_d2_ are the Doppler shifts from two channels of train-borne dual-channel DRS. Therefore, although the deflection angle Δ*φ* is dynamically variable, the train-borne dual-channel DRS can accurately calculate the travel speed *v* and the deflection angle Δ*φ* by solving Equation (3).

By comparing Equation (3) with Equation (2), it can be found that the dual-channel DRS with the Janus configuration is a special case of dual-channel DRS where *f*_1_ = *f*_2_ and *φ*_1_ + *φ*_2_ = *π*. Thus, the vehicle-borne dual-channel DRS with the Janus configuration is the most simplified structure of a train-borne dual-channel DRS. The main advantage of train-borne dual-channel DRS is the suppression of the multipath effect shown in Figure 3b. The train-borne dual-channel DRS makes use of the different emitted frequencies *f*_1_ and *f*_2_, which can provide frequency diversity to avoid the signal interference problem of two channels as well as different design incident angles *φ*_1_ and *φ*_2_, which can provide spatial diversity and generate different Doppler shifts over multiple paths. The frequency and spatial diversity reduce the probability of the multipath effect, and solve the specular multipath problems more effectively [20,21]. Second, the train-borne dual-channel DRS seeks no approximation methods for the solution of train speed calculation according to Equation (3), and it has no restriction requirements of the deflection angle Δ*φ*. Therefore, it has a higher speed-measuring accuracy than the vehicle-borne dual-channel DRS with the Janus configuration. Finally, since the train-borne dual-channel DRS adopts the single-sided asymmetric structure and its two antennas of dual-channel point in the same direction, it can determine the travel direction of the train, depending on whether two Doppler shifts of its dual-channel are positive or negative [18,19].

## 3. Speed Calibration

CTCS-3 has extremely strict requirements on train operation safety and speed control. In the high-speed railway passenger dedicated lines that are greater than or equal to 300 km/h, the speed controls of the train-borne speed measurement subsystem are specified as sounding an alarm at an overspeed of 2 km/h, triggering the service brake at an overspeed of 5 km/h, and triggering the emergency brake at an overspeed of 15 km/h, according to the safety requirements of CTCS-3.

Since CTCS-3 has a high safety requirement of train speed control, the speed-measuring accuracy of all speed sensors used in the train-borne speed measurement subsystem must be assured by calibration to ensure the accuracy, reliability, and traceability of the train-borne speed measurement results. Conventional train-borne wheel speed sensors can be calibrated by using standard equipment for revolution speed [22], which can be traced to the source of the national standard for the revolution speed of China. However, the calibration method and traceability of train-borne DRS are still an ongoing issue that has been unsolved completely thus far.

### 3.1. Traditional Calibration Methods

There are two primary traditional methods that have been used for train-borne DRS speed calibration: the acoustic resonant method based on the Doppler shift simulation by using tuning forks, and the mechanical movement method based on the relative motion principle by using a moving pavement simulator.

#### 3.1.1. Tuning Fork

**Figure 6:**
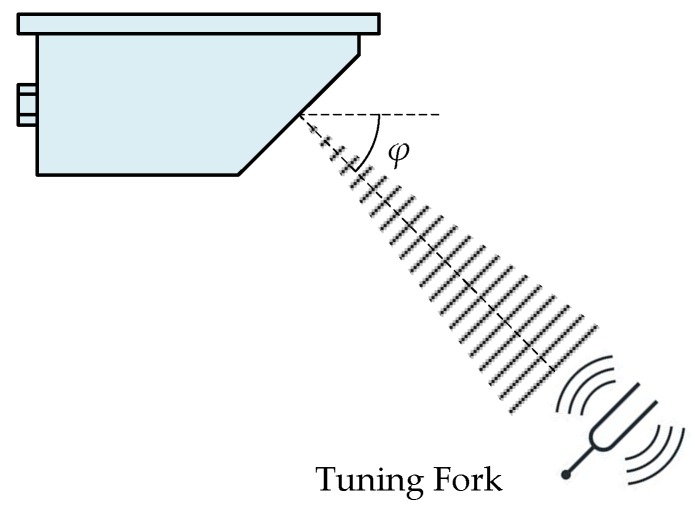
Schematic diagram of the traditional speed calibration method for a train-borne single-channel DRS by using tuning forks.

Tuning forks have been widely used for calibrating traffic radar-based speed meters [13], CW radar sensors for distance, and Doppler frequency measurements [14], etc. The schematic diagram of the speed calibration method for a train-borne single-channel DRS by using tuning forks is shown in Figure 6. The tuning fork, with a specific acoustic resonant frequency *f*_TF_ that is equal to the Doppler shift frequency *f*_d_ according to Equation (1), is struck and then placed in front of the transceiver antenna of the train-borne single-channel DRS to be calibrated to simulate a moving rail surface with a specific speed *v*. The detailed calibration process of the train-borne single-channel DRS is similar to that of the speed enforcement down-the-road radar, which has been discussed in detail in [13].

However, the traditional speed calibration method by using tuning forks is only applicable to the train-borne single-channel DRS, and not to the train-borne dual-channel DRS due to the problem of acoustic resonant signal interference when two tuning forks are struck simultaneously. Another disadvantage of this calibration method is the discontinuous problem of speed calibration points, which means the low resolution of the simulated speed. The specific acoustic resonant frequency *f*_TF_ is the natural frequency of the corresponding tuning fork, which means that the simulated Doppler shift *f*_d_ corresponds to a tuning fork. Therefore, if any speed calibration points are desired, a large number of tuning forks will be required in the Doppler shift simulator using the acoustic resonant approach.

#### 3.1.2. Moving Pavement Simulator

**Figure 7:**
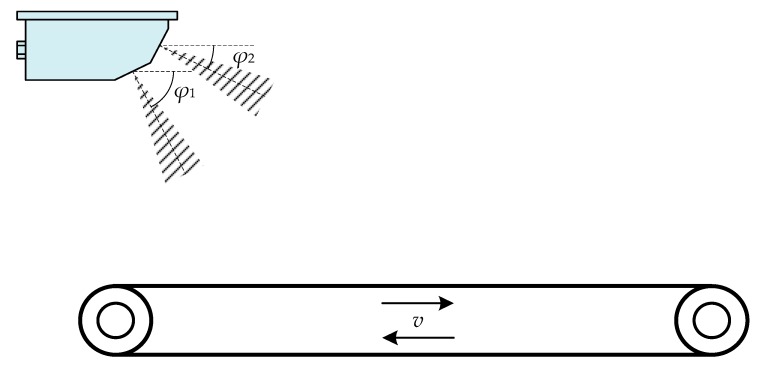
Schematic diagram of traditional speed calibration method for the train-borne DRS by using a moving pavement simulator.

Another traditional speed calibration method for a train-borne DRS is based on the principle of relative motion by using a moving pavement simulator, as shown in Figure 7 [15,16]. The moving pavement simulator is composed of a motor, a driving wheel, a driven wheel, a mesh belt, a controller, an encoder, etc. The train-borne DRS to be calibrated is mounted directly above the moving pavement simulator. The mesh belt of the simulator moves at the intended linear speed value *v*, converted from the rotational speed of the driving wheel and the driven one, driven by the motor and controlled by the controller. According to the relative motion principle, the above speed calibration method is equivalent to a train with the train-borne DRS to be calibrated moving along the railway at a speed value *v*.

Since the above speed calibration method simulates the work scene of a train-borne DRS by using the moving pavement simulator in a mechanical movement approach, it is applicable to the calibration of a single-channel DRS as well as a dual-channel DRS. However, there are two main disadvantages of this traditional calibration method. The first disadvantage is the limited speed simulation range, which is only up to 50 km/h due to the restrictions of the system performance and personal safety, while the speed-measuring upper range of the rain-borne single-channel DRS is usually up to 250 km/h, and that of the train-borne dual-channel DRS is up to 500 km/h or even 600 km/h. This results in the fact that this traditional calibration method cannot meet the calibration requirements of the train-borne DRS in full range. Another disadvantage is the traceability problem and performance evaluation of the moving pavement simulator. The speed-measuring accuracy of the train-borne single-channel DRS is within the range of ±1 km/h at a speed value less than 100 km/h and ±1 % at a speed value greater than or equal to 100 km/h while that of the train-borne dual-channel DRS is within the range of ±0.5 km/h at a speed value less than 100 km/h and ±0.5 % at a speed value greater than or equal to 100 km/h. It has a high requirement for speed simulation accuracy and performance of the moving pavement simulator used for calibrating train-borne DRSs. However, there has been no progress in tracing and evaluating the moving pavement simulator presently, and therefore, the uncertainty of the calibration results on train-borne DRS has not been evaluated until now.

### 3.2. New Calibration Method and Process

In order to ensure the accuracy, reliability, and traceability of train-borne DRS speed measurements in full range, a new speed calibration method for a train-borne DRS was proposed based on the Doppler shift signal simulation approach realized by establishing a programmable and composable 24 GHz CW radar target simulation system for a train-borne DRS in this paper, which is applicable for the calibration of single-channel DRSs as well as dual-channel DRSs.

#### 3.2.1. Calibration Method

The new speed calibration method is also based on the Doppler shift simulation principle, which is similar to that of the traditional speed calibration method by using tuning forks. However, the realization of the new method is not by using the acoustic resonant approach, but by imposing a signal modulation on the incident CW microwave signal by using a moving target simulator.

**Figure 8:**
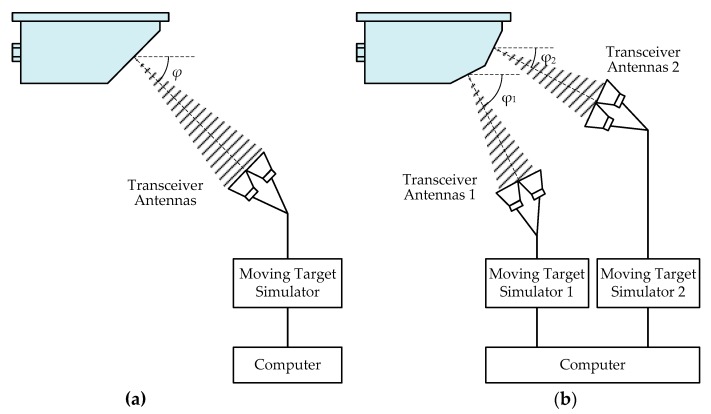
Schematic diagram of the proposed speed calibration method: (**a**) For the train-borne single-channel DRS; (**b**) For the train-borne dual-channel DRS.

The schematic diagram of the proposed speed calibration method is shown in Figure 8, where (a) is for the train-borne single-channel DRS and (b) is for the train-borne dual-channel DRS. As shown in Figure 8a, since the train-borne single-channel DRS has only one incident CW microwave signal with the emitted frequency *f*_0_ at the design incident angle *φ*, there is only one moving target simulator required for its speed calibration, while there are two moving target simulators required for the speed calibration of train-borne dual-channel DRS because it has two incident CW microwave signals with the different emitted frequencies of *f*_1_ and *f*_2_ at the respective design incident angles of *φ*_1_ and *φ*_2_, as shown in Figure 8b. By comparing Figure 8b with Figure 8a, it can be found that the new speed calibration method for the train-borne single-channel DRS is a simplified case of that for the train-borne dual-channel DRS, and the moving target simulator can be shared by calibration for the single-channel DRS as well as dual-channel DRS. Therefore, this section focuses on the principle analysis of the new speed calibration method for the train-borne dual-channel DRS, which is similar but more complicated than that for the train-borne single-channel DRS.

As shown in Figure 8b, the dual-channel DRS to be calibrated is placed on a stable platform. Since the train-borne dual-channel DRS has two incident CW microwave signals with different emitted frequencies of *f*_1_ and *f*_2_ at the respective design incident angles of *φ*_1_ and *φ*_2_, two moving target simulators are required for its speed calibration. The transceiver antennas of the first moving target simulator are aimed at the first antenna of the dual-channel DRS with the design incident angle *φ*_1_ while those of the second moving target simulator are aimed at the second antenna with the design incident angle *φ*_2_. Transceiver antennas of the moving target simulator include a receiving antenna to receive the CW microwave signals from the dual-channel DRS to be calibrated and a transmitting antenna to transmit the Doppler echo signals back to it. The moving target simulator is similar to a Doppler shift signal generator that provides Doppler echo signals with a Doppler shift frequency *f*_d_ that corresponds to a specific speed value *v* for a given emitted frequency *f*_0_. A program-controlled computer with software is used for entering the simulated speed value *v* with motion direction, and setting the technical parameters of dual-channel DRS to be calibrated, mainly including the design incident angles *φ*_1_ and *φ*_2_, emitted frequencies *f*_1_ and *f*_2_, etc.

#### 3.2.2. Calibration Process

**Figure 9:**
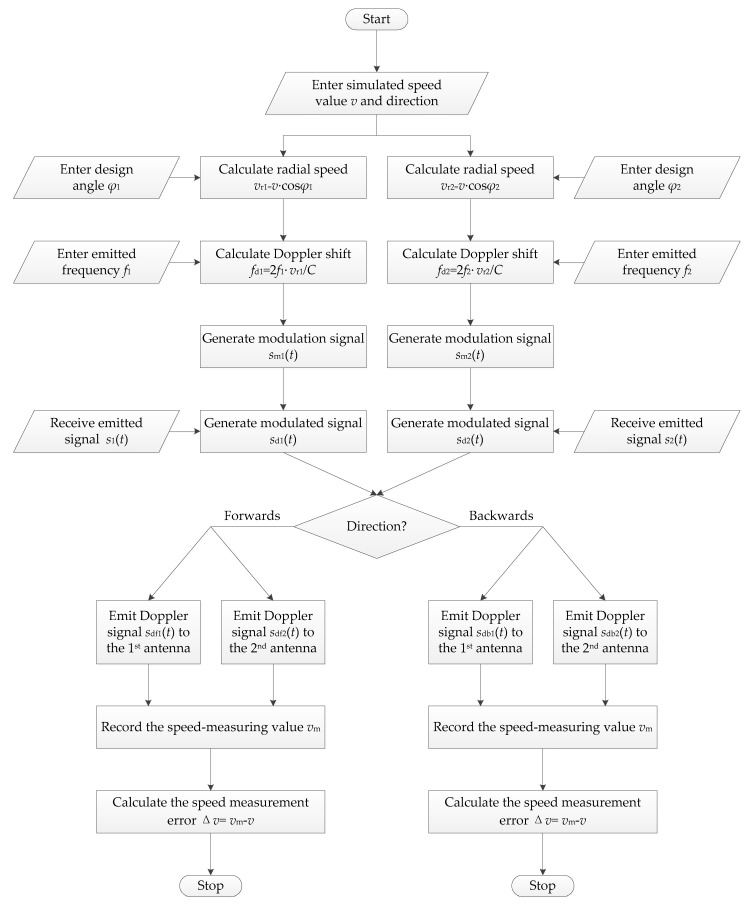
Flow diagram of the new speed calibration process for a dual-channel DRS.

The flow diagram of the new speed calibration process for a dual-channel DRS is given in Figure 9. According to the input simulated speed value *v*, forward or backward motion direction, and technical parameters of the dual-channel DRS, the program-controlled computer with software first calculates two Doppler shift frequencies *f*_d1_ and *f*_d2_, corresponding to two channels of the dual-channel DRS to be calibrated based on the Doppler shift formula, and are given by Equation (4):

(4) $$\{\begin{array}{c} f_{d1}=\frac{2}{C}\cdot f_{1}\cdot v\cdot \cos\phi_{1}\\ f_{d2}=\frac{2}{C}\cdot f_{2}\cdot v\cdot \cos\phi_{2} \end{array}.$$

Next, the computer sends the value and sign of *f*_d1_ to the first moving target simulator and sends those of *f*_d2_ to the second moving target simulator through the cables simultaneously.

After receiving two Doppler shift frequencies *f*_d1_ and *f*_d2_ from the program-controlled computer with software, the two moving target simulators respectively generate two modulation signals *s*_m1_(*t*) and *s*_m2_(*t*), which are expressed by Equation (5):

(5) $$\{\begin{array}{c} s_{m1}\left(t\right)=A_{m1}\cdot \cos\left(2\pi f_{d1}t+\alpha_{m1}\right)\\ s_{m2}\left(t\right)=A_{m2}\cdot \cos\left(2\pi f_{d2}t+\alpha_{m2}\right) \end{array},$$

where *A*_m1_ and *A*_m2_ represent the amplitudes of two modulation signals; *α*_m1_ and *α*_m2_ represent the phases; and *t* represents time.

Meanwhile, the transceiver antennas of the two moving target simulators respectively receive two CW microwave signals *s*_1_(*t*) and *s*_2_(*t*) with different emitted frequencies of *f*_1_ and *f*_2_ transmitted from two antennas of the dual-channel DRS to be calibrated at the respective design incident angles of *φ*_1_ and *φ*_2_. Two emitted signals *s*_1_(*t*) and *s*_2_(*t*) are expressed by Equation (6):

(6) $$\{\begin{array}{c} s_{1}\left(t\right)=A_{1}\cdot \cos\left(2\pi f_{1}t+\alpha_{1}\right)\\ s_{2}\left(t\right)=A_{2}\cdot \cos\left(2\pi f_{2}t+\alpha_{2}\right) \end{array},$$

where *A*_1_ and *A*_2_ represent the initial amplitudes of two emitted signals, and *α*_1_ and *α*_2_ represent the initial phases.

After receiving two emitted signals and generating two modulation signals, the two moving target simulators respectively impose amplitude modulations on two incident emitted signals by multiplying two modulation signals, and generate two modulated signals *s*_d1_(*t*) and *s*_d2_(*t*), which are expressed by Equation (7):

(7) $$\{\begin{array}{c} s_{d1}\left(t\right)=s_{\mathrm{df}1}\left(t\right)+s_{\mathrm{db}1}\left(t\right)\\ s_{d2}\left(t\right)=s_{\mathrm{df}2}\left(t\right)+s_{\mathrm{db}2}\left(t\right) \end{array},$$

where

(8) $$\{\begin{array}{c} s_{\mathrm{df}1}\left(t\right)=\frac{A_{1}\cdot A_{m1}}{2}\cdot \cos\left[2\pi \left(f_{1}+f_{d1}\right)t+\left(\phi_{1}+\phi_{m1}\right)\right]\\ s_{\mathrm{df}2}\left(t\right)=\frac{A_{2}\cdot A_{m2}}{2}\cdot \cos\left[2\pi \left(f_{2}+f_{d2}\right)t+\left(\phi_{2}+\phi_{m2}\right)\right] \end{array},$$

and

(9) $$\{\begin{array}{c} s_{\mathrm{db}1}\left(t\right)=\frac{A_{1}\cdot A_{m1}}{2}\cdot \cos\left[2\pi \left(f_{1}-f_{d1}\right)t+\left(\phi_{1}-\phi_{m1}\right)\right]\\ s_{\mathrm{db}2}\left(t\right)=\frac{A_{2}\cdot A_{m2}}{2}\cdot \cos\left[2\pi \left(f_{2}-f_{d2}\right)t+\left(\phi_{2}-\phi_{m2}\right)\right] \end{array}.$$

According to the input simulated motion direction, the two moving target simulators generate two Doppler signals after filtering two modulated signals *s*_d1_(*t*) and *s*_d2_(*t*). When the forward motion direction is chosen, two Doppler signals generated by two moving target simulators are *s*_df1_(*t*) and *s*_df2_(*t*) as given in Equation (8), where the frequencies of two Doppler signals *s*_df1_(*t*) and *s*_df2_(*t*) have been shifted to (*f*_1_ + *f*_d1_) and (*f*_2_ + *f*_d2_). When the backward motion direction is chosen, two Doppler signals that are generated by two moving target simulators are changed to *s*_db1_(*t*) and *s*_db2_(*t*) as given in Equation (9), where the frequencies of two Doppler signals *s*_db1_(*t*) and *s*_db2_(*t*) have been shifted to (*f*_1_ – *f*_d1_) and (*f*_2_ – *f*_d2_). Compared with the wo incident CW microwave signals transmitted from the dual-channel DRS to be calibrated, the two Doppler signals generated by two moving target simulators are identical to the echo signals received by two channels of the dual-channel DRS to be calibrated moving forwards or backwards at the speed value *v*.

Finally, two Doppler signals are respectively retransmitted to two antennas of the dual-channel DRS to be calibrated by the transceiver antennas of the two moving target simulators. Then, the dual-channel DRS to be calibrated processes the received two Doppler signals to measure the simulated speed. The measured value of the simulated speed *v*_m_ is recorded to calculate the simulated speed measurement error Δ*v*, which is expressed by Equation (10):

(10) $$\Delta v=v_{m}-v.$$

The above new speed calibration method by using the moving target simulator is also applicable to the train-borne single-channel DRS. The speed calibration process for a single-channel DRS is similar to that for the dual-channel DRS as above-mentioned, but only one moving target simulator is required to impose the modulation on only one incident emitted signal of the single-channel DRS by multiplying one corresponding modulation signal to generate the corresponding Doppler signal.

#### 3.2.3. Advantage Analysis

First, the new speed calibration method has the advantage of a high resolution of the simulated speed points. The resolution of the simulated speed point is decided by the resolution of the Doppler shift frequency generated by the moving target simulator. This is similar to a Doppler shift signal generator, which provides an echo signal with a Doppler shift frequency *f*_d_ corresponding to a specific speed value *v* and a given emitted frequency *f*_0_. Therefore, it is not difficult for the moving target simulator to generate a Doppler shift frequency with a resolution 0.1 Hz or up to 0.01 Hz if required, which means that the resolution of the simulated speed point will be 0.001 km/h or up to 0.0001 km/h, if required. 

Second, another advantage of the new speed calibration method is the wide simulated speed range. The simulated speed range is also decided by the range of the Doppler shift frequency generated by a moving target simulator, and is also not difficult for the moving target simulator to generate a Doppler shift frequency with a range up to 2 kHz, which means that the maximum value of the simulated speed point will be up to 500 km/h. Therefore, it can meet the speed calibration requirements of a train-borne DRS in full range.

Finally, the simulated speed of the new speed calibration method can be traced to the Doppler shift frequency generated by the moving target simulator, so the speed simulation accuracy and performance of the moving target simulator can be evaluated to make calibration results more convincing, which will be discussed in the next two sections.

## 4. Realization and Traceability

To verify the proposed speed calibration method by using the moving target simulator, a 24 GHz CW radar target simulation system for a train-borne DRS (abbreviated to “simulation system” hereafter) was specially designed with a flexible and composable scheme to calibrate a single-channel DRS as well as a dual-channel DRS. Furthermore, the portable design of the simulation system could meet the calibration requirements of the train-borne DRS in the laboratory as well as in the field, while the programmable design satisfied the requirements of calibrating various types of train-borne DRSs with different technical parameters at any speed points as desired. Since the simulated speed accuracy is decided by the accuracy of the Doppler shift frequency generated by the moving target simulator, the traceability and performance evaluation of the simulated speed were taken into account in the simulation system by measuring the Doppler shift frequency corresponding to the simulated speed value.

### 4.1. Realization of the Simulation System

**Figure 10:**
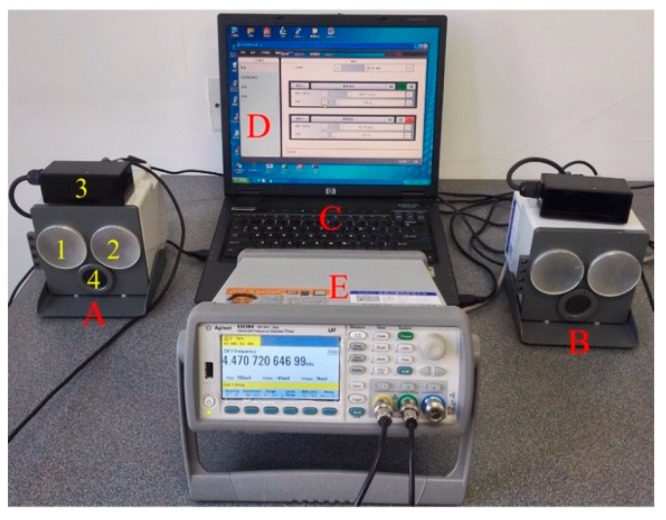
Realization and traceability of the simulation system.

The simulation system is realized as shown in Figure 10, which adopted a portable design to calibrate train-borne DRSs in the laboratory as well as in the field. The simulation system included two identical moving target simulators (marked as ‘A’ and ‘B’ in Figure 10) and a laptop (marked as ‘C’ in Figure 10) equipped with the control software (marked as ‘D’ in Figure 10). Two moving target simulators had the same technical parameters. Using the moving target simulator A as an example to explain its composition: ‘1′ represents its transmitted antenna, ‘2′ represents its received antenna, ‘3′ is a frequency converter to send the generated Doppler shift frequency to a dual-channel universal frequency counter (marked as ‘E’ in Figure 10) for traceability, and ‘4′ is an aiming device used to aim at the antenna of the train-borne DRS. When the simulation system is used for calibrating a single-channel DRS, only one moving target simulator and the laptop with software are required for speed calibration and only one channel of the dual-channel universal frequency counter is required for measuring the generated Doppler shift frequency used for simulated speed traceability. When the simulation system is used for calibrating a dual-channel DRS, both moving target simulators and the laptop with software are required for speed calibration and both channels of the dual-channel universal frequency counter are required for the simulated speed traceability.

The main parameters of the simulation system are listed in Table 1. To meet the speed calibration requirement of the train-borne DRS with high accuracy, the maximum permissible error (MPE) of the simulated speed of the simulation system was designed to be ±0.05 km/h. The simulated Doppler shift frequency range of the simulation system was up to (10~18,000) Hz and the simulated emitted frequency range was from 24,050 MHz to 24,250 MHz. Therefore, the simulated radial speed range of the simulation system was up to (1~400) km/h when the simulated incident angle was set to 0°. When the simulated incident angle range was limited to (45 ± 8)°, the simulated speed range of the simulation system could reach (5~500) km/h to satisfy the speed calibration requirement of the train-borne DRS in full range.

### 4.2. Traceability

Referring to China’s national verification regulation of test equipment for vehicle speed radar measurement meters [23], the simulated speed of the simulation system can be traced to the Doppler shift frequency measured by the dual-channel universal frequency counter (marked as ‘E’ in Figure 10). The simulated speed error of the simulation system can be evaluated by the Doppler shift frequency, which is generated by the moving target simulator in three aspects: accuracy, stability, and fluctuation [23].

#### 4.2.1. Accuracy

The simulated speed error component Δ*V*_1_ in the accuracy aspect can be evaluated by the Doppler shift frequency error Δ*f*_d_ between the measured value *f*_dm_ and the theoretical one *f*_d0_, where Δ*f*_d_ = *f*_dm_ – *f*_d0_. According to the simulated radial speed *V* and the emitted frequency *f*_0_ intended to be simulated by the moving target simulator, the theoretical value *f*_d0_ of the Doppler shift frequency is calculated based on the Doppler shift formula, and the measured value *f*_dm_ of the Doppler shift frequency generated by the moving target simulator can be measured by the dual-channel universal frequency counter. Then, the simulated speed error component Δ*V*_1_ in the accuracy aspect can be calculated by Equation (11) [23]:

(11) $$\Delta V_{1}=\frac{\Delta f_{d}}{f_{d0}}\cdot V.$$

Since the nominal values of the two emitted frequencies of typical train-borne dual-channel DRS products are mostly 24,060 MHz and 24,190 MHz, the simulated emitted frequency of the first moving target simulator was set to 24,190 MHz and that of the 2^nd^ moving target simulator was set to 24,060 MHz. Table 2 shows the Doppler shift frequency error Δ*f*_d_ and the corresponding simulated speed error component Δ*V*_1_ of the first moving target simulator at the simulated radial speed points *V* of 1.000 km/h, 10.000 km/h, 60.000 km/h, 100.000 km/h, 200.000 km/h, 300.000 km/h, and 400.000 km/h, respectively. Table 3 shows those of the second moving target simulator at the same simulated radial speed points *V* with Table 2.

#### 4.2.2. Stability

The simulated speed error component Δ*V*_2_ in the stability aspect can be evaluated by the frequency stability of the Doppler shift generated by the moving target simulator. According to the recommended testing method in [23], a total of 101 consecutive measured values of Doppler shift frequency generated by the moving target simulator are required to calculate the frequency stability *σ*, where the frequency measurement gate-time of the dual-channel universal frequency counter is set to 1 s. The frequency stability *σ* can be calculated by Equation (12) [23]:

(12) $$\sigma =\frac{1}{f_{d0}}\cdot \sqrt{\sum_{i=1}^{I}\frac{{\left(f_{\mathrm{dm}}^{i+1}-f_{\mathrm{dm}}^{i}\right)}^{2}}{2I}},$$

where $f_{\mathrm{dm}}^{i}$ denotes the *i*^th^ measured value of the Doppler shift frequency; $f_{\mathrm{dm}}^{i+1}$ denotes the *i* + 1^th^ measured value, and *I* = 100. Then, the simulated speed error component Δ*V*_2_ in the stability aspect can be calculated by Equation (13) [23]:

(13) $$\Delta V_{2}=2\gamma \cdot \sigma \cdot V,$$

where γ = 2/3 because a crystal oscillator is used in the moving target simulator [23].

Table 4 shows the frequency stability *σ* of the Doppler shift and the corresponding simulated speed error component Δ*V*_2_ of the first moving target simulator with the same simulated parameters as Table 2, while Table 5 shows those of the second moving target simulator with the same simulated parameters as Table 3.

#### 4.2.3. Fluctuation

The simulated speed error component Δ*V*_3_ in the fluctuation aspect can be evaluated by the frequency fluctuation of the Doppler shift generated by the moving target simulator. According to the recommended testing method in [23], the 13 measured values of the Doppler shift frequency generated by the moving target simulator are measured by the dual-channel universal frequency counter every 5 min in 1 h, where the frequency measurement gate-time is set to 10 s. The maximum measured value $f_{\mathrm{dm}}^{\max}$ and the minimum one $f_{\mathrm{dm}}^{\min}$ of the 13 measured values are found to calculate the frequency fluctuation *S* by Equation (14) [23]:

(14) $$S=\frac{f_{\mathrm{dm}}^{\max}-f_{\mathrm{dm}}^{\min}}{f_{d0}}.$$

Then, the simulated speed error component Δ*V*_3_ in the fluctuation aspect can be calculated by Equation (15) [23]:

(15) $$\Delta V_{3}=S\cdot V.$$

Table 6 shows the frequency fluctuation *S* of the Doppler shift and the corresponding simulated speed error component Δ*V*_3_ of the first moving target simulator with the same simulated parameters as Table 2, while Table 7 shows those of the second moving target simulator with the same simulated parameters as Table 3.

#### 4.2.4. Simulated Speed Error of the Simulation System

Referring to the recommended evaluation method in [23], the simulated speed error Δ*V* of moving target simulator can be calculated by Equation (16):

(16) $$\Delta V=\sqrt{\Delta V_{1}^{2}+\Delta V_{2}^{2}+\Delta V_{3}^{2}}.$$

Table 8 and Table 9 show the calculation results of the simulated speed error Δ*V* of two moving target simulators, respectively. It can be seen from Table 8 and Table 9 that the simulated speed errors of the two moving target simulators were both less than 0.05 km/h, which can verify the simulated speed MPE of the simulation system given in Table 1.

## 5. Calibration Results and Uncertainty Evaluation

To validate the effectiveness and feasibility of the proposed speed calibration method, two typical train-borne dual-channel DRS samples were chosen to be calibrated in full range by using the realized simulation system to evaluate its technical performance in this section. The two types of samples were both DRS05 manufactured by DEUTA-WERKE GmbH, but the subtypes were different, namely DRS05/1a and DRS05S1c. A DRS05/1a sample (Serial Number: 15039067 /B) was chosen to be calibrated in the laboratory with controlled environmental conditions and a DRS05S1c sample (Serial Number: 11037430 /A) was chosen to be calibrated in the field with uncontrolled environmental conditions.

### 5.1. Calibration Results of DRS05/1a in the Laboratory

DRS05/1a is mainly used for the speed measurement of urban rail trains in the railway freight dedicated lines of urban, suburban, and intercity areas. The calibration of a DRS05/1a sample was performed in the laboratory with the temperature range of (23 ± 5) °C and humidity lower than 80% RH, and without vibration and electromagnetic interference.

**Figure 11:**
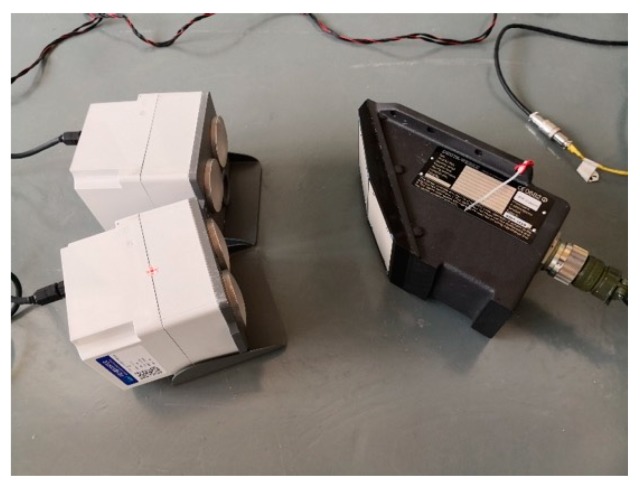
Calibration setup for the DRS05/1a sample.

The calibration setup for the DRS05/1a sample is shown in Figure 11. According to the specifications for the DRS05/1a sample, the design incident angle of its first channel is 50° and the nominal emitted frequency of that is 24,190 MHz, while the design incident angle of its second channel is 40° and the nominal emitted frequency is 24,060 MHz. The calibration parameters of the simulation system were set according to the design parameters of the DRS05/1a sample. For the first moving target simulator, the simulated emitted frequency was set to 24,190 MHz and the simulated incident angle was set to 50°. For the second moving target simulator, the simulated emitted frequency was set to 24,060 MHz and the simulated incident angle was set to 40°. The transceiver antennas of the first moving target simulator were aimed at the first antenna of DRS05/1a, while the antennas of the second moving target simulator were aimed at the second antenna of DRS05/1a at the same time. The simulated speed points were set to several typical values from 5.00 km/h to 500.00 km/h through the control software on the laptop.

Table 10 shows the numerical calibration results of the DRS05/1a sample at various simulated speed points. The average of ten independent speed measurement values was taken as the measured value of the simulated speed to calculate the simulated speed measurement error and relative error. It can be seen from Table 10 that all of the relative errors at the above simulated speed points were within the range of ±0.5% in full range of (5~500) km/h and the repeatability of ten independent measurement values was quite good, validating that the technical performance of the simulation system and the new speed calibration method by using the moving target simulator are an effective calibration method for train-borne dual-channel DRSs in the laboratory with controlled environmental conditions.

### 5.2. Calibration Results of DRS05S1c in the Field

DRS05S1c has an integrated protective cover against the impact of stones and extreme temperatures, and is especially suitable for measuring the speed of high-speed trains in high-speed railway passenger dedicated lines. The calibration of a DRS05S1c sample was performed in the field with uncontrolled environmental conditions.

**Figure 12:**
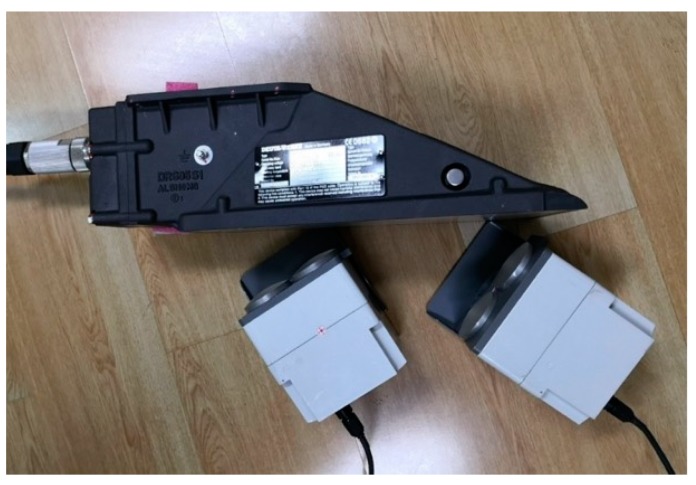
Calibration setup for the DRS05S1c sample.

The calibration setup for the DRS05S1c sample is shown in Figure 12. According to the specifications, DRS05S1c has the same technical parameters as DRS05/1a, and therefore the calibration parameters of the simulation system were setup the same as those in Section 5.1. Compared with DRS05/1a, DRS05S1c has an integrated protective cover at the bottom, and it cannot determine the location of the two channels of DRS05S1c by visual observation and the aiming device of the moving target simulator. Therefore, it needed to adjust the positions of the two moving target simulators according to the amplitudes of the incident CW microwave signals received from the two channels of DRS05S1c before calibration, and ensured that the transceiver antennas of the two moving target simulators are aimed at the two corresponding channels of DRS05S1c at the same time.

The simulated speed points of the DRS05S1c sample were the same as those of the DRS05/1a sample within the speed range of (5.00~500.00) km/h. Table 11 shows the numerical calibration results of the DRS05S1c sample at various simulated speed points. The calibration results performed ten independent speed measurements at every simulated speed point, and took the average value of ten independent speed measurement values as the measured value of the simulated speed to calculate the simulated speed measurement error and relative error. It can be seen from Table 11 that all the relative errors of the DRS05S1c sample at the simulated speed points were also within the range of ±0.5% in full range of (5~500) km/h. However, the repeatability of ten independent measurement values was not as good as that of the DRS05/1a sample at high speed points. This was partly caused by the uncontrolled and unknown environmental conditions. Another important reason was the aiming error between the transceiver antennas of the moving target simulator and the corresponding channel of DRS05S1c.

### 5.3. Uncertainty Evaluation

Referring to the international standard for the uncertainty of measurement [24] and China’s national standard for the evaluation and expression of uncertainty in measurement [25], the uncertainty of the speed calibration results in Section 5.1 and Section 5.2 is evaluated in this subsection.

In the uncertainty evaluation for speed calibration, the mathematical model for the speed measurement error Δ*v* is given in Equation (10). Therefore, the uncertainty model can be expressed by Equation (17):

(17) $$u_{c}^{2}\left(\Delta v\right)=c_{1}^{2}\cdot u^{2}\left(v_{m}\right)+c_{2}^{2}\cdot u^{2}\left(v\right),$$

where *u*_c_(Δ*v*) is the standard uncertainty of the speed measurement error Δ*v*; *u*(*v*_m_) is the standard uncertainty component associated with the measured value of simulated speed *v*_m_; *u*(*v*) is the standard uncertainty component associated with the reference value of simulated speed *v*; and *c*_1_ and *c*_2_ are the sensitivity coefficients, which equal to

(18) $$\begin{array}{cc} c_{1}=\frac{\partial \Delta v}{\partial v_{m}}=1 & c_{2}=\frac{\partial \Delta v}{\partial v}=-1 \end{array}.$$

Therefore, the standard uncertainty *u*_c_(Δ*v*) in Equation (17) can be simplified as

(19) $$u_{c}\left(\Delta v\right)=\sqrt{u^{2}\left(v_{m}\right)+u^{2}\left(v\right)}.$$

The standard uncertainty component *u*(*v*_m_) associated with the measured value of the simulated speed *v*_m_ includes two sub-components *u*_1_(*v*_m_) and *u*_2_(*v*_m_). *u*_1_(*v*_m_) is the standard uncertainty sub-component associated with the repeatability of the ten independent speed measurement values in Table 10 and Table 11, and can be calculated by the Bessel formula:

(20) $$s\left(x_{n}\right)=\sqrt{\frac{1}{M-1}\sum_{j=1}^{M}{\left(x_{n}^{j}-\bar{x_{n}}\right)}^{2}},$$

where *x_n_* denotes the reference value of the *n*^th^ simulated speed point; $x_{n}^{j}$ denotes the *j*^th^ measurement value of the *n*^th^ simulated speed point; ${\bar{x}}_{n}$ denotes the average value of ten independent measurement values of the *n*^th^ simulated speed point; and *M* = 10. Since the average value of ten independent speed measurement values was taken as the measured value of the simulated speed to calculate the simulated speed measurement error, the standard uncertainty sub-component associated with the speed measurement repeatability can be calculated by Equations (20) and (21):

(21) $$u_{1}\left(v_{m}\right)=s\left(\bar{x_{n}}\right)=s\left(x_{n}\right)/\sqrt{M}.$$

where *u*_2_(*v*_m_) is the standard uncertainty sub-component associated with the speed measurement resolution of DRS05/1a and DRS05S1c, which is 0.1 km/h, as shown in Table 10 and Table 11, and therefore can be calculated by Equation (22):

(22) $$u_{2}\left(v_{m}\right)=\frac{0.1}{2\sqrt{3}}=0.029\mathrm{km}/h.$$

Since the two sub-components *u*_1_(*v*_m_) and *u*_2_(*v*_m_) are independent and uncorrelated, the standard uncertainty component *u*(*v*_m_) can be calculated as

(23) $$u\left(v_{m}\right)=\sqrt{u_{1}^{2}\left(v_{m}\right)+u_{2}^{2}\left(v_{m}\right)}.$$

The standard uncertainty component *u*(*v*) associated with the reference value of the simulated speed *v* depends on the simulated speed error of the simulation system. According to the technical parameters of the simulation system given in Table 1, the MPE of the simulated speed was ±0.05 km/h and is estimated by rectangular distribution. Then, the standard uncertainty component *u*(*v*) can be calculated as

(24) $$u\left(v\right)=\frac{0.05}{\sqrt{3}}=0.029\mathrm{km}/h.$$

According to the calculation results of Equations (23) and (24), the standard uncertainty *u*_c_(Δ*v*) can be calculated by Equation (19). The expanded uncertainty *U*(Δ*v*) of the speed measurement error Δ*v*, where the coverage factor *k* is 2, can be expressed by Equation (25):

(25) $$\begin{array}{cc} U\left(\Delta v\right)=k\cdot u_{c}\left(\Delta v\right) & \left(k=2\right) \end{array}.$$

Based on the above analysis of the uncertainty evaluation and the speed calibration results of two train-borne dual-channel DRS samples given in Table 10 and Table 11, the uncertainty evaluation results of the two DRS samples were respectively calculated and given in Table 12 and Table 13. For the speed calibration results of the DRS05/1a sample, the maximum value of the expanded uncertainty of the speed measurement error was 0.09 km/h, as shown in Table 12. For the speed calibration results of the DRS05S1c sample, the maximum value of the expanded uncertainty was 0.12 km/h, as shown in Table 13. Therefore, the expanded uncertainty of the DRS05S1c calibration results was larger than that of the DRS05/1a calibration results, which means that the calibration results of DRS05/1a were more accurate than those of DRS05S1c. The main reason for this is that the repeatability of ten independent speed measurements of DRS05/1a was better than that of DRS05S1c. Furthermore, the expanded uncertainty values of the two DRS samples were both less than one third of the simulated speed MPE of the simulation system given in Table 1, and therefore the speed calibration results of the two DRS samples given in Table 10 and Table 11 were both effective and reliable [24,25].

## 6. Conclusions and Future Work

A new speed calibration process and traceability for train-borne DRSs before being mounted on the train are proposed in this paper. The architecture and working principle of train-borne DRSs with different structures were first introduced. Then, the disadvantages of two traditional speed calibration methods for train-borne DRS were described, and a new speed calibration method based on the Doppler shift signal simulation approach was proposed. The details of the proposed speed calibration process were illustrated and the techniques on the design of the moving target simulator were presented. A simulation system was specifically realized to verify the proposed speed calibration method for train-borne DRSs, and the traceability and performance evaluation on the simulated speed were taken into account. When the simulated incident angle range was within the range of (45 ± 8) °, the simulated speed range of the simulation system was up to (5~500) km/h and the MPE of the simulated speed was ±0.05 km/h, which satisfies the speed calibration requirements of train-borne DRSs in full range. Finally, the calibration results and uncertainty evaluation results of two typical train-borne dual-channel DRS samples validated the effectiveness and feasibility of the proposed speed calibration approach for train-borne DRSs with full range in the laboratory as well as in the field.

For further work, we will conduct further study on in situ speed calibration for train-borne DRSs after being mounted on the train, especially online speed calibration when the train is in motion. In situ and online speed calibration have the advantage of the overall study of possible factors affecting the actual speed measurement results such as the antenna pattern shape of the DRS, disturbance from flying stones created by the train movement, the multipath effect caused by multi-reflection by concrete sleepers on subgrade or stones on the ballasted track, distance between the DRS and subgrade, etc.

## Figures and Tables

**Table 1:** Main parameters of the simulation system.

Parameter	Value
Simulated emitted frequency range	(24,050~24,250) MHz
Simulated Doppler shift frequency range	(10~18,000) Hz
Simulated incident angle range	(45 ± 8) °
Simulated speed range	(5~500) km/h
MPE of simulated speed	±0.05 km/h

**Table 2:** Doppler shift frequency error and simulated speed error component in the accuracy aspect of the first moving target simulator at emitted frequency of 24,190 MHz.

Simulated Radial Speed *V* (km/h)	Doppler Shift Frequency			Simulated Speed Error Component Δ*V*_1_ (km/h)
	Theoretical Value *f*_d0_ (Hz)	Measured Value *f*_dm_ (Hz)	Frequency Error Δ*f*_d_ (Hz)	
1.000	44.8273	44.6773	−0.1500	−0.003
10.000	448.2731	448.2374	−0.0357	−0.001
60.000	2689.6385	2690.1568	0.5183	0.012
100.000	4482.7308	4483.1063	0.3755	0.008
200.000	8965.4616	8964.7476	−0.7140	−0.016
300.000	13,448.1924	13,447.8537	−0.3387	−0.008
400.000	17,930.9233	17,930.2272	−0.6961	−0.016

**Table 3:** Doppler shift frequency error and simulated speed error component in the accuracy aspect of the second moving target simulator at emitted frequency of 24,060 MHz.

Simulated Radial Speed *V* (km/h)	Doppler Shift Frequency			Simulated Speed Error Component Δ*V*_1_ (km/h)
	Theoretical Value *f*_d0_ (Hz)	Measured Value *f*_dm_ (Hz)	Frequency Error Δ*f*_d_ (Hz)	
1.000	44.5864	44.6773	0.0909	0.002
10.000	445.8640	446.0402	0.1762	0.004
60.000	2675.1840	2674.7761	−0.4079	−0.009
100.000	4458.6401	4458.9366	0.2965	0.007
200.000	8917.2801	8916.4083	−0.8718	−0.020
300.000	13,375.9202	13,375.3447	−0.5755	−0.013
400.000	17,834.5603	17,833.5486	−1.0117	−0.023

**Table 4:** Doppler shift frequency stability and simulated speed error component in the stability aspect of the first moving target simulator at emitted frequency of 24,190 MHz.

Simulated Radial Speed *V* (km/h)	Doppler Shift Frequency		Simulated Speed Error Component Δ*V*_2_ (km/h)
	Theoretical Value *f*_d0_ (Hz)	Stability *σ* (×10^-8^)	
1.000	44.8273	39.7	0.000001
10.000	448.2731	5.3	0.000001
60.000	2689.6385	4.5	0.000004
100.000	4482.7308	2.8	0.000004
200.000	8965.4616	2.2	0.000006
300.000	13,448.1924	1.7	0.000007
400.000	17,930.9233	1.5	0.000008

**Table 5:** Doppler shift frequency stability and simulated speed error component in the stability aspect of the second moving target simulator at emitted frequency of 24,060 MHz.

Simulated Radial Speed *V* (km/h)	Doppler Shift Frequency		Simulated Speed Error Component Δ*V*_2_ (km/h)
	Theoretical Value *f*_d0_ (Hz)	Stability *σ* (×10^-8^)	
1.000	44.5864	38.2	0.000001
10.000	445.8640	5.1	0.000001
60.000	2675.1840	4.7	0.000004
100.000	4458.6401	3.0	0.000004
200.000	8917.2801	2.5	0.000007
300.000	13,375.9202	1.9	0.000008
400.000	17,834.5603	1.6	0.000009

**Table 6:** Doppler shift frequency fluctuation and simulated speed error component in the fluctuation aspect of the first moving target simulator at emitted frequency of 24,190 MHz.

Simulated Radial Speed *V* (km/h)	Doppler Shift Frequency		Simulated Speed Error Component Δ*V*_3_ (km/h)
	Theoretical Value *f*_d0_ (Hz)	Fluctuation *S* (×10^-7^)	
1.000	44.8273	22.3	0.000002
10.000	448.2731	8.9	0.00001
60.000	2689.6385	9.3	0.00006
100.000	4482.7308	9.1	0.00009
200.000	8965.4616	8.9	0.00018
300.000	13,448.1924	8.9	0.00027
400.000	17,930.9233	8.8	0.00035

**Table 7:** Doppler shift frequency fluctuation and simulated speed error component in the fluctuation aspect of the second moving target simulator at emitted frequency of 24,060 MHz.

Simulated Radial Speed *V* (km/h)	Doppler Shift Frequency		Simulated Speed Error Component Δ*V*_3_ (km/h)
	Theoretical Value *f*_d0_ (Hz)	Fluctuation *S* (×10^-7^)	
1.000	44.5864	22.4	0.000002
10.000	445.8640	11.2	0.00001
60.000	2675.1840	9.3	0.00006
100.000	4458.6401	9.0	0.00009
200.000	8917.2801	9.0	0.00018
300.000	13,375.9202	8.9	0.00027
400.000	17,834.5603	8.8	0.00035

**Table 8:** Simulated speed error of the first moving target simulator.

Simulated Radial Speed *V* (km/h)	Simulated Speed Error Component			Simulated Speed Error Δ*V* (km/h)
	Accuracy Δ*V*_1_ (km/h)	Stability Δ*V*_2_ (km/h)	Fluctuation Δ*V*_3_ (km/h)	
1.000	−0.003	0.000001	0.000002	0.003
10.000	−0.001	0.000001	0.00001	0.001
60.000	0.012	0.000004	0.00006	0.012
100.000	0.008	0.000004	0.00009	0.008
200.000	−0.016	0.000006	0.00018	0.016
300.000	−0.008	0.000007	0.00027	0.008
400.000	−0.016	0.000008	0.00035	0.016

**Table 9:** Simulated speed error of the second moving target simulator.

Simulated Radial Speed *V* (km/h)	Simulated Speed Error Component			Simulated Speed Error Δ*V* (km/h)
	Accuracy Δ*V*_1_ (km/h)	Stability Δ*V*_2_ (km/h)	Fluctuation Δ*V*_3_ (km/h)	
1.000	0.002	0.000001	0.000002	0.002
10.000	0.004	0.000001	0.00001	0.004
60.000	−0.009	0.000004	0.00006	0.009
100.000	0.007	0.000004	0.00009	0.007
200.000	−0.020	0.000007	0.00018	0.020
300.000	−0.013	0.000008	0.00027	0.013
400.000	−0.023	0.000009	0.00035	0.023

**Table 10:** Numerical speed calibration results of the DRS05/1a sample.

Reference Value of Simulated Speed *v* (km/h)	Speed Measurement Value (km/h)					Measured Value *v*_m_ (km/h)	Error Δ*v* (km/h)	Relative Error (%)
	1	2	3	4	5			
5.00	5.0	5.0	5.1	5.0	5.0	5.02	0.02	0.40
	5.0	5.0	5.1	5.0	5.0			
10.00	10.0	10.0	9.9	10.0	10.0	9.98	−0.02	−0.20
	10.0	10.0	9.9	10.0	10.0			
20.00	20.0	20.0	20.0	20.0	20.0	20.00	0.00	0.00
	20.0	20.0	20.0	20.0	20.0			
40.00	39.9	39.9	39.9	39.9	39.9	39.90	−0.10	−0.25
	39.9	39.9	39.9	39.9	39.9			
60.00	59.9	59.9	59.9	59.9	59.9	59.90	−0.10	−0.17
	59.9	59.9	59.9	59.9	59.9			
80.00	79.8	79.8	79.8	79.8	79.8	79.80	−0.20	−0.25
	79.8	79.8	79.8	79.8	79.8			
100.00	99.8	99.8	99.8	99.8	99.8	99.80	−0.20	−0.20
	99.8	99.8	99.8	99.8	99.8			
120.00	119.8	119.8	119.8	119.8	119.8	119.80	−0.20	−0.17
	119.8	119.8	119.8	119.8	119.8			
150.00	149.8	149.8	149.8	149.8	149.8	149.80	−0.20	−0.13
	149.8	149.8	149.8	149.8	149.8			
180.00	179.9	179.9	179.9	179.9	179.9	179.90	−0.10	−0.06
	179.9	179.9	179.9	179.9	179.9			
200.00	199.9	199.9	199.9	199.9	199.9	199.90	−0.10	−0.05
	199.9	199.9	199.9	199.9	199.9			
250.00	249.8	249.8	249.8	249.8	249.8	249.80	−0.20	−0.08
	249.8	249.8	249.8	249.8	249.8			
300.00	300.1	300.1	300.1	300.1	300.1	300.10	0.10	0.03
	300.1	300.1	300.1	300.1	300.1			
400.00	400.4	400.4	400.4	400.4	400.4	400.40	0.40	0.10
	400.4	400.4	400.4	400.4	400.4			
500.00	500.9	500.9	500.9	500.9	500.9	500.90	0.90	0.18
	500.9	500.9	500.9	500.9	500.9			

**Table 11:** Numerical speed calibration results of the DRS05S1c sample.

Reference Value of Simulated Speed *v* (km/h)	Speed Measurement Value (km/h)					Measured Value *v*_m_ (km/h)	Error Δ*v* (km/h)	Relative Error (%)
	1	2	3	4	5			
5.00	5.0	5.0	5.0	5.0	5.0	5.00	0.00	0.00
	5.0	5.0	5.0	5.0	5.0			
10.00	10.0	10.0	10.0	10.0	10.0	10.00	0.00	0.00
	10.0	10.0	10.0	10.0	10.0			
20.00	19.9	19.9	19.9	19.9	19.9	19.90	−0.10	−0.50
	19.9	19.9	19.9	19.9	19.9			
40.00	39.8	39.8	39.8	39.8	39.8	39.80	−0.20	−0.50
	39.8	39.8	39.8	39.8	39.8			
60.00	59.9	59.9	59.9	59.9	59.9	59.90	−0.10	−0.17
	59.9	59.9	59.9	59.9	59.9			
80.00	79.8	79.8	79.8	79.8	79.8	79.80	−0.20	−0.25
	79.8	79.8	79.8	79.8	79.8			
100.00	99.7	99.7	99.7	99.7	99.7	99.70	−0.30	−0.30
	99.7	99.7	99.7	99.7	99.7			
120.00	119.6	119.6	119.6	119.6	119.6	119.60	−0.40	−0.33
	119.6	119.6	119.6	119.6	119.6			
150.00	149.6	149.6	149.7	149.6	149.7	149.64	−0.36	−0.24
	149.6	149.6	149.7	149.6	149.7			
180.00	179.5	179.5	179.5	179.6	179.6	179.54	−0.46	−0.26
	179.5	179.5	179.5	179.6	179.6			
200.00	199.5	199.5	199.5	199.6	199.6	199.54	−0.46	−0.23
	199.5	199.5	199.5	199.6	199.6			
250.00	249.7	249.6	249.5	249.8	249.7	249.66	−0.34	−0.14
	249.5	249.7	249.6	249.7	249.8			
300.00	299.6	299.7	299.8	299.5	299.7	299.66	−0.34	−0.11
	299.5	299.8	299.7	299.6	299.7			
400.00	399.1	399.2	399.0	399.1	399.1	399.10	−0.90	−0.23
	399.0	399.1	399.1	399.1	399.2			
500.00	499.5	499.4	499.6	499.8	499.6	499.58	−0.42	−0.08
	499.6	499.6	499.8	499.4	499.5			

**Table 12:** Uncertainty evaluation results of the DRS05/1a sample.

Reference Value of Simulated Speed *v* (km/h)	Measured Value *v*_m_ (km/h)	Error Δ*v* (km/h)	*u*(*v*_m_) (km/h)			*u*(*v*) (km/h)	*u*_c_(Δ*v*) (km/h)	*U*(Δ*v*) (km/h)
			*u*_1_(*v*_m_)	*u*_2_(*v*_m_)	*u*(*v*_m_)			
5.00	5.02	0.02	0.013	0.029	0.032	0.029	0.043	0.09
10.00	9.98	−0.02	0.013	0.029	0.032	0.029	0.043	0.09
20.00	20.00	0.00	0.000	0.029	0.029	0.029	0.041	0.08
40.00	39.90	−0.10	0.000	0.029	0.029	0.029	0.041	0.08
60.00	59.90	−0.10	0.000	0.029	0.029	0.029	0.041	0.08
80.00	79.80	−0.20	0.000	0.029	0.029	0.029	0.041	0.08
100.00	99.80	−0.20	0.000	0.029	0.029	0.029	0.041	0.08
120.00	119.80	−0.20	0.000	0.029	0.029	0.029	0.041	0.08
150.00	149.80	−0.20	0.000	0.029	0.029	0.029	0.041	0.08
180.00	179.90	−0.10	0.000	0.029	0.029	0.029	0.041	0.08
200.00	199.90	−0.10	0.000	0.029	0.029	0.029	0.041	0.08
250.00	249.80	−0.20	0.000	0.029	0.029	0.029	0.041	0.08
300.00	300.10	0.10	0.000	0.029	0.029	0.029	0.041	0.08
400.00	400.40	0.40	0.000	0.029	0.029	0.029	0.041	0.08
500.00	500.90	0.90	0.000	0.029	0.029	0.029	0.041	0.08

**Table 13:** Uncertainty evaluation results of the DRS05S1c sample.

Reference Value of Simulated Speed *v* (km/h)	Measured Value *v*_m_ (km/h)	Error Δ*v* (km/h)	*u*(*v*_m_) (km/h)			*u*(*v*) (km/h)	*u*_c_(Δ*v*) (km/h)	*U*(Δ*v*) (km/h)
			*u*_1_(*v*_m_)	*u*_2_(*v*_m_)	*u*(*v*_m_)			
5.00	5.00	0.00	0.000	0.029	0.029	0.029	0.041	0.08
10.00	10.00	0.00	0.000	0.029	0.029	0.029	0.041	0.08
20.00	19.90	−0.10	0.000	0.029	0.029	0.029	0.041	0.08
40.00	39.80	−0.20	0.000	0.029	0.029	0.029	0.041	0.08
60.00	59.90	−0.10	0.000	0.029	0.029	0.029	0.041	0.08
80.00	79.80	−0.20	0.000	0.029	0.029	0.029	0.041	0.08
100.00	99.70	−0.30	0.000	0.029	0.029	0.029	0.041	0.08
120.00	119.60	−0.40	0.000	0.029	0.029	0.029	0.041	0.08
150.00	149.64	−0.36	0.016	0.029	0.033	0.029	0.044	0.09
180.00	179.54	−0.46	0.016	0.029	0.033	0.029	0.044	0.09
200.00	199.54	−0.46	0.016	0.029	0.033	0.029	0.044	0.09
250.00	249.66	−0.34	0.034	0.029	0.045	0.029	0.053	0.11
300.00	299.66	−0.34	0.034	0.029	0.045	0.029	0.053	0.11
400.00	399.10	−0.90	0.021	0.029	0.036	0.029	0.046	0.09
500.00	499.58	−0.42	0.044	0.029	0.053	0.029	0.060	0.12
